# The Breast Imaging Reporting and Data System (BI-RADS) in the Dutch breast cancer screening programme: its role as an assessment and stratification tool

**DOI:** 10.1007/s00330-012-2409-2

**Published:** 2012-03-14

**Authors:** J. M. H. Timmers, H. J. van Doorne-Nagtegaal, H. M. Zonderland, H. van Tinteren, O. Visser, A. L. M. Verbeek, G. J. den Heeten, M. J. M. Broeders

**Affiliations:** 1 National Expert and Training Centre for Breast Cancer Screening, PO Box 6873, 6503 GJ Nijmegen, the Netherlands; 2 Department of Epidemiology, Biostatistics and HTA, Radboud University Nijmegen Medical Centre, PO Box 9101, 6500 HB Nijmegen, The Netherlands; 3 Comprehensive Cancer Centre The Netherlands (IKNL), location Amsterdam, PO Box 9236, 1006 AE Amsterdam, The Netherlands; 4 Department of Radiology, Academic Medical Centre, University of Amsterdam, PO Box 22660, 1100 DD Amsterdam, The Netherlands

**Keywords:** BI-RADS, Positive predictive value, Quality assessment, Performance, Mammographic screening

## Abstract

**Objectives:**

To assess the suitability of the Breast Imaging Reporting and Data System (BI-RADS) as a quality assessment tool in the Dutch breast cancer screening programme.

**Methods:**

The data of 93,793 screened women in the Amsterdam screening region (November 2005–July 2006) were reviewed. BI-RADS categories, work-up, age, final diagnosis and final TNM classification were available from the screening registry. Interval cancers were obtained through linkage with the cancer registry. BI-RADS was introduced as a pilot in the Amsterdam region before the nationwide introduction of digital mammography (2009–2010).

**Results:**

A total of 1,559 women were referred to hospital (referral rate 1.7 %). Breast cancer was diagnosed in 485 women (detection rate 0.52 %); 253 interval cancers were reported, yielding a programme sensitivity of 66 % and specificity of 99 %. BI-RADS 0 had a lower positive predictive value (PPV, 14.1 %) than BI-RADS 4 (39.1 %) and BI-RADS 5 (92.9 %; *P* < 0.0001). The number of invasive procedures and tumour size also differed significantly between BI-RADS categories (*P* < 0.0001).

**Conclusion:**

The significant differences in PPV, invasive procedures and tumour size match with stratification into BI-RADS categories. It revealed inter-observer variability between screening radiologists and can thus be used as a quality assessment tool in screening and as a stratification tool in diagnostic work-up.

**Key Points:**

• *The BI-RADS atlas is widely used in breast cancer screening programmes.*

• *There were significant differences in results amongst different BI-RADS categories.*

• *Those differences represented the radiologists’ degree of suspicion for malignancy, thus enabling stratification of referrals.*

• *BI-RADS can be used as a quality assessment tool in screening.*

• *Training should create more uniformity in applying the BI-RADS lexicon.*

## Introduction

The Dutch nationwide breast cancer screening programme was introduced in 1989 and fully implemented in 1997. Originally, the target population consisted of women aged 50–69 but in 1998 the upper age limit was raised to 75 [1]. Every 2 years, eligible women are invited for mammographic screening. Since the start of the programme, over 13 million screening examinations have been performed and 56,000 breast cancers have been detected [2]. In The Netherlands, women with a positive screening examination are referred to their general practitioner and subsequently to a hospital of their choice for further assessment [3]. Diagnostic work-up after referral is not an integrated part of the screening programme like in many other countries but is carried out in general or academic hospitals (a reason to avoid the more common international term ‘recall’). Additional imaging (e.g. ultrasound and/or magnification views) is also performed in these hospitals and only when necessary, additional imaging is followed by a biopsy.

The National Expert and Training Centre for Breast Cancer Screening (NETCB) aims to safeguard and constantly improve the quality of the breast cancer screening programme [4]. Recurrent site visits are performed as part of the national quality assurance programme. During these site visits, the NETCB observed cases of miscommunication between screening radiologists, general practitioners and hospital specialists. As a result, the national guideline “breast cancer” [3] recommended the use of the Breast Imaging Reporting and Data System (BI-RADS) [5] in the screening programme and this was made compulsory by the National Institute for Public Health and the Environment (RIVM) in 2008. The RIVM coordinates and directs the population screening programmes on behalf of the Ministry of Public Health, Welfare and Sports (VWS).

The BI-RADS atlas is developed by the American College of Radiology (ACR) to improve communication between physicians, and provides standardised mammographic reporting, breast imaging terminology, a report organisation and a classification system [5]. It also provides a complete follow-up and outcome monitoring system that allows a screening or clinical practice to determine performance outcomes such as the positive predictive value (PPV) and the percentage of small and node negative cancers. These quality assurance data are meant to improve the quality of patient care [5]. Several studies have shown that the use of BI-RADS in a clinical setting can be useful in predicting the presence of malignancy and improving the choice and efficiency of further necessary examinations [6–9]. It has been widely adopted in clinical practice throughout the world. BI-RADS is also implemented in screening programmes in the United States [10] and Europe [11, 12].

We retrospectively assessed the introduction of BI-RADS in the Amsterdam screening region, a pilot study before the nationwide introduction. The purpose of this study was to evaluate its feasibility, inter-observer agreement and its ability to stratify referred women according to the chance of malignancy, tumour size and follow-up procedures. Furthermore, we identified interval cancers to assess the overall performance of the screening programme of the Amsterdam region.

## Materials and methods

### Study population

The data of 93,793 women (aged 49–75) who participated in the programme of the Amsterdam screening region (between November 2005 and July 2006) were reviewed. Women consented for the anonymous use of their data for research. The mammograms are independently read by two certified screening radiologists who must reach consensus about referral for further diagnostic assessment. If consensus is not reached, a third radiologist will decide. The result of a screening mammogram is classified as “suspicious” (these women are referred for diagnostic work-up in a hospital) or “not suspicious” (no diagnostic work-up is necessary).

### Assessment and diagnosis

In most hospitals the mammograms of the referred women were repeated and completed with ultrasound and/or magnification views and, if necessary, biopsies were performed. Information on each individual woman (age at diagnosis, BI-RADS category, final TNM classification [UICC 2002], the type of diagnostic work-up and final diagnosis) was derived from the screening registry and cancer registry.

True-positive final diagnosis is defined as a carcinoma found within 1 year after referral in the screening programme. When there is no carcinoma found within one year after a positive screening examination, it is called a false-positive screening examination [1, 5]. The cases with a TP diagnosis were classified as ductal carcinoma in situ (DCIS), <1 cm, ≥1 cm and X (indefinite).

### Interval cancers

To assess the overall performance of the screening programme and each unit separately (programme sensitivity and specificity), we identified the number of interval cancers by linking the screening registry to the regional cancer registry. Interval cancers are breast cancers diagnosed in women within 2 years after a negative screening examination. Positive matches were manually checked to exclude screen-detected cases of a later screening round [13].

### Radiologists

In the Amsterdam screening region, four groups of screening radiologists (screenings units) are responsible for the evaluation of the mammographic examinations. On average, a screening radiologist reads over 12,000 screening mammograms per year. Screening radiologists did not receive a dedicated training programme on how to use BI-RADS in the screening programme. However, all of them were familiar with the use of BI-RADS in a clinical setting.

### BI-RADS

The ACR guidelines [5] define a negative screening examination as one that is negative or has benign findings (BI-RADS categories 1 and 2) and a positive screening examination as one for which referral is initiated (BI-RADS categories 0, 4 or 5; Table 1). BI-RADS 3 was initially included in this pilot study. Although this category is negative, it suggests short interval follow-up and this is not available in the Dutch screening setting. As suggested by the ACR 2003 guidelines [5], this category was excluded at the final nationwide introduction of BI-RADS in the screening. In this study, the group of 0 and 3 were reviewed and was found to represent a normal mix of abnormalities with low suspicion requiring additional (imaging) work-up. They were therefore merged into BI-RADS 0.

**Table 1:** BI-RADS categories used in the Dutch screening setting

Assessment category	Description	Referral to hospital
0	Possible finding, need for additional imaging information	Yes
1	Negative	No
2	Benign finding(s)	No
4	Suspicious abnormality, not the classic appearance for malignancy, but has a reasonable possibility of being malignant	Yes
5	Highly suggestive of malignancy	Yes

### Data analysis

To calculate the PPV of the BI-RADS categories, we used the number of TP diagnoses among the total of referrals per category × 100 %. Logistic regression analysis was used to estimate the influence of age together with BI-RADS outcome on the PPV. Variation in PVV among screening units was also taken into consideration. Data were analysed with the statistical package R (version 2.8.1) [14]. Results were expressed in terms of odds ratios (OR) with 95 % confidence intervals (CI) and *P* values. The ACR defines sensitivity as the probability of detecting a cancer when a cancer exists and specificity as the probability of interpreting an examination as negative when cancer does not exist [5].

## Results

### Study population

Between 1 November 2005 and 30 June 2006, 93,793 women participated in the screening programme for breast cancer in the Amsterdam region and 1,559 women were referred to a hospital for diagnostic work-up (referral rate 1.7 %). Thirty-four women were referred for possible bilateral breast cancers; therefore the total number of breasts referred in this period was 1,593. Data on eight referrals without final diagnosis were excluded from the analysis. Another 39 referrals were excluded because the BI-RADS classification of the screening examination was not available. One case was retrospectively classified as a technical failure with extreme underexposure on one side in a very large breast. This case was therefore excluded from further analysis, leaving 1,545 referred breasts in 1,511 women in the analysis.

All referred women went to hospital for diagnostic work-up; 357 (23 %) referrals were first screens, while 1,189 (76 %) came from subsequent screens. The average age of the women was 59 (SD 7.7). Women in the first screening cycle were on average 10 years younger than the overall age of woman in subsequent screens.

### Screening performance

The programme sensitivity was 66 % (95 % CI 63–69), based on 485 true positives and 253 false negatives. The programme specificity was 99 % (95 % CI 99–99), based on 91,995 true negatives and 1,060 false positives (Table 2). The programme sensitivity of the screening units ranged between 62-68 %, whereas the programme specificity of the screening units was more than 99 % for all.

**Table 2:** Screening outcomes of the screening programme in the Amsterdam region (The Netherlands)

	Breast cancer (*n*)	No breast cancer (*n*)	Total	Predictive value (95 % CI)
Referral (*n*)	485 (TP)	1,060 (FP)	1,545	PPV = 31 % (29–34)
No referral (*n*)	253 (FN)	91,995 (TN)	92,248	NPV = 99 % (99–99)
Total	738	93,055	93,793	
	Sensitivity = 66 %	Specificity = 99 %		

*TP* true positive screening examination, *FP* false positive screening examination, *FN* false negative screening examination, *TN* true negative screening examination, *FN* false negative screening examination, *TN* true negative screening examination, *PPV* positive predictive value, *NPV* negative predictive value

Sensitivity = 485/(485 + 253) = 66 % (95 % CI: 63-69), specificity = 91,995/(1,060 + 91,995) = 99 % (95 % CI 99-99)

### BI-RADS, breast cancer and follow-up

Of the 1,545 referrals, 811 were assigned BI-RADS category 0 (53 %), 578 BI-RADS category 4 (37 %) and 156 BI-RADS category 5 (10 %) (Table 3). Of the referrals, 485 (31 %) were true positives and 1,060 (69 %) were false positives. The PPV of BI-RADS 0 was 14 % (95 % CI: 12–17), of BI-RADS 4 39 % (95 % CI 35–43) and of BI-RADS 5 93 % (95 % CI 88–96) (Table 3).

**Table 3:** Diagnostic work-up and tumour size according to BI-RADS category

BI-RADS	*n*	Breast cancer (*n*)		PPV (%)	Pathology (%)	DCIS (%)	<1 cm (%)	≥1 cm (%)	Imaging only (%)
		Screen detected	Interval cancers						
0	811	114	-	14.1	384 (47.3)	26 (22.8)	43 (37.7)	45 (39.5)	427 (52.7)
4	578	226	-	39.1	366 (63.3)	43 (19.0)	78 (34.5)	105 (46.5)	212 (36.7)
5	156	145	-	92.9	149 (95.5)	6 (4.2)	26 (17.9)	113 (77.9)	7 (4.5)
Total	1,545	485	-	31.4	899 (58.2)	75 (15.5)	147 (30.3)	263 (54.2)	646 (41.8)
1/2	92,247	-	253	-	-	20 (7.9)	33 (13.0)	200 (79.1)	-

Table 3 also shows the tumour size of cases with a true-positive diagnosis. Tumour size was positively related with the BI-RADS category. Although the positive predictive value of BI-RADS 0 was relatively low, this group still accounted for the highest number (38 %) of smaller tumours (<1 cm), while 78 % of the BI-RADS 5 tumours were larger than 1 cm.

Diagnostic work-up differed for each BI-RADS category. The most invasive measures were performed in BI-RADS 5 (96 %) (Table 3). A biopsy procedure was performed in 47 % of the women with BI-RADS 0 and 53 % of the women with BI-RADS 0 received only non-invasive imaging procedures.

### Factors associated with the PPV of BI-RADS

**Fig. 1:**
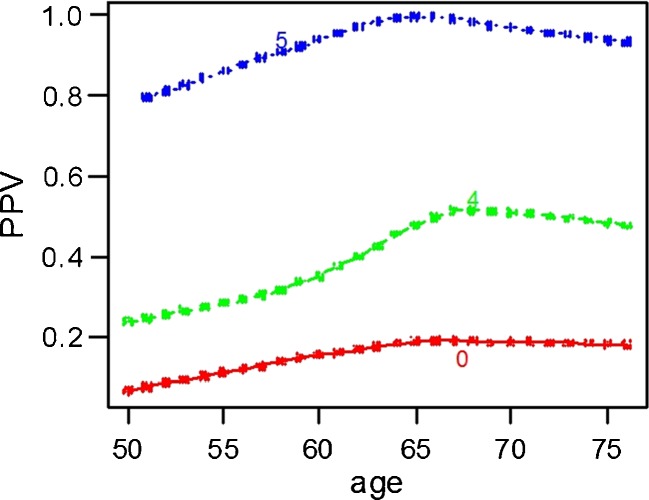
Relation between age and BI-RADS and PPV

The PPV of BI-RADS increased with increasing age (see Table 4). There is also a relationship between age and the probability of having a TP assessment, stratified by BI-RADS category. This is shown in Fig. 1 (non-parametric regression [LOESS] estimates).

**Table 4:** The influence of BI-RADS and age on positive predictive value, expressed as odds ratios from logistic regression analysis

	*n* ^a^	PPV (%)^b^	OR	(CI 95 %)
BI-RADS category				
0	811	114 (14.1)	1	-
4	578	226 (39.1)	4.6	(3.4-6.2)
5	156	145 (92.9)	93	(47–183)
Age				
50-54	528	104 (19.7)	1	-
55-59	307	84 (27.4)	1.4	(0.94-2.1)
60-67	396	154 (39.6)	2.4	(1.7-3.5)
68-76	314	143 (45.5)	3.1	(2.2-4.6)

*OR* odds ratio, *CI* confidence interval, *PPV* positive predictive value

^a^ Total number = 1,545

^b^ Total number = 484

Another factor associated with the PPV is the significant difference (*P* < 0.001) in assigning BI-RADS categories between the four screening units (Table 5). Assigning BI-RADS 0 varied from 27 % to 72 % and BI-RADS 4 from 20 % to 62 %, while BI-RADS 5 showed little difference (from 8 % to 14 % of the cases).

**Table 5:** BI-RADS categories and PPV by screening unit

Screening unit	Screening examination *n* (%)	Positive examination *n* (%)	BI-RADS 0 *n* (%)	TP (PPV)	BI-RADS 4 *n* (%)	TP (PPV)	BI-RADS 5 *n* (%)	TP (PPV)	FN *n*	Programme sensitivity % (95 % CI)	Specificity % (95 % CI)
1	28,138	476 (30.1)	127 (26.7)	16 (12.6)	297 (62.4)	88 (29.6)	52 (10.9)	48 (92.3)	70	68 (62-74)	99 (99-99)
2	18,758	308 (20.0)	186 (60.4)	20 (10.8)	94 (30.5)	34 (36.2)	28 ( 9.1)	27 (96.4)	50	62 (54-70)	99 (99-99)
3	1,8758	257 (16.6)	137 (53.3)	27 (19.7)	85 (33.1)	43 (50.6)	35 (13.6)	33 (94.3)	58	64 (56-71)	99 (99-99)
4	28,138	504 (32.6)	361 (71.6)	51 (14.1)	102 (20.2)	61 (59.8)	41 (8.1)	37 (90.2)	75	66 (60-72)	99 (99-99)
Total	93,793	1,545 (100)	811 (52.5)	114 (14.1)	578 (37.4)	226 (39.1)	156 (10.1)	145 (92.9)	253	66 (63-96)	99 (99-99)

*TP* true positive screening examination, *FN* false negative screening examination

## Discussion

In this retrospective study we evaluated the use of the BI-RADS final assessment in the Dutch breast cancer screening setting. The differences in PPV, invasive procedures and tumour size between the BI-RADS categories confirm that stratification of the referred cases ties in with the radiologist’s level of suspicion. The number of more subtle and favourable tumours in the BI-RADS 0 category stresses the importance of referring also the less suspicious and more subtle abnormalities to hospital for further assessment. At the same time the assignment of the BI-RADS categories revealed significant differences between screening units in this study.

The difference in BI-RADS application between units did not result in differences in interval cancers. The overall performance of the Amsterdam screening region is somewhat lower than the national screening programme (71 % until 2004 [1]) which may be explained by the fact that Amsterdam is an urban region [15]. Because of a higher number of referrals and the introduction of digital mammography, the detection rate in The Netherlands increased every year from 4.9 in 2005 to 5.7 in 2009. The effect on the number of interval carcinomas will become evident in the next couple of years. Until the introduction of BI-RADS in the screening, the screening radiologists received regular feedback on their performance on a local basis. As peer review and outcome monitoring will increase the quality of patient care [5], the outcomes of all women referred and interval carcinomas detected were periodically evaluated. However, this system of outcome monitoring did not reveal the inter-observer variability between the four screening units and inconsistency in applying the BI-RADS lexicon, in our study mainly BI-RADS 0 and 4. Only in the relatively small group of BI-RADS 5 did we find, as expected, a substantial agreement. We also found that in larger numbers of screening examinations, BI-RADS is able to stratify the referred women adequately.

Several other studies [16–18] also showed a substantial inter-observer variation in assigning the BI-RADS categories. This can possibly be explained by a lack of dedicated BI-RADS training and local variation in its application. Even expert radiologists have limited consistency in using the BI-RADS assessment categories [19], but according to Berg [20] proper training and continuing application may improve this. As the preliminary results of this study became available early, the NETBC developed a dedicated BI-RADS training programme for all screening radiologists. This training programme significantly improved the inter-observer variation among new screening radiologists [21]. Training will also create more uniformity in applying the BI-RADS lexicon by the screening radiologists as described in several studies [20, 22, 23].

The introduction of the BI-RADS in the screening setting functions as an interesting benchmark indicator in our new continuous medical quality audit, especially on a local level it revealed significant differences between units. Therefore we believe that BI-RADS can be used as a quality assessment tool for screening radiologists.

Consistent use of BI-RADS is also of importance to those who are involved in the diagnostic work-up. Screening mammography is effective for the early detection of breast cancer. However, most referred cases prove to be benign after further assessment either with only imaging (additional views and ultrasound) or with additional biopsy procedures. In our study, more than half of the women with BI-RADS 0 could be dismissed after only imaging procedures at the radiology department. Instead, they visited the multidisciplinary team which is mandatory according to our national breast cancer guidelines [3]. As BI-RADS differentiates between cases with a high (BI-RADS 4 or 5) or low suspicion (BI-RADS 0) for malignancy, it provides knowledge of the chance of malignancy and can thus determine the urgency and type of further assessment. The BI-RADS 0 cases could then be assessed separately from the BI-RADS 4 and 5 cases, for instance along a “quick assessment route” to the hospital radiology departments. Policy makers should be aware of the possibilities of BI-RADS as a stratification tool and consider reviewing the current policies as it could possibly reduce waiting times, costs and unnecessary anxiety [24].

There are several factors that might have influenced the results of this study. Firstly, BI-RADS categories 0, 4 and 5 in our study are assigned on the basis of a screening mammogram only. Thus, they represent no final findings and further imaging work-up still needs to be performed. This might have been reflected in a different mix of BI-RADS 0 and 4 for the referred cases in our study compared with studies in the United States or other European countries [10, 11]. This decision how to perform the diagnostic work-up is the responsibility of the clinical radiologist who is not necessarily the same person as the screening radiologist. Secondly, international comparison is difficult due to additional differences in characteristics of the screening programmes, including the criteria for referral. The referral rate in The Netherlands is still among the lowest worldwide [25–27]. Therefore, comparing our results with studies from the United States or other countries in Europe is therefore not straightforward; it has led to differences in the PPV of BI-RADS in our results compared with the PPV in other studies. Taplin [10] described a PPV of BI-RADS category 4 of 16.7 % (versus 39 % in our study) and a PPV of BI-RADS category 5 of 68.4 % (versus 93 % in our study). Our results were comparable with those from another study from The Netherlands, that reported a PPV of BI-RADS category 4 of 40 % and a BI-RADS category 5 of 100 % in a screening population [6].

In conclusion, the significant differences we found in PPV, invasive procedures and tumour size match with the stratification into BI-RADS categories and thus represent the radiologists’ degree of suspicion for malignancy. BI-RADS can therefore be used as a quality assessment tool in the screening and stratification tool in the diagnostic work-up. Training of screening radiologists should create more uniformity in the application of BI-RADS.
